# Limbal Stem Cell Transplantation: Clinical Results, Limits, and Perspectives

**DOI:** 10.1155/2018/8086269

**Published:** 2018-10-11

**Authors:** Marta Sacchetti, Paolo Rama, Alice Bruscolini, Alessandro Lambiase

**Affiliations:** ^1^Department of Sense Organs, Sapienza University of Rome, Rome, Italy; ^2^Cornea and Ocular Surface Unit, San Raffaele Hospital, Milan, Italy

## Abstract

Limbal stem cell deficiency (LSCD) is a clinical condition characterized by damage of cornea limbal stem cells, which results in an impairment of corneal epithelium turnover and in an invasion of the cornea by the conjunctival epithelium. In these patients, the conjunctivalization of the cornea is associated with visual impairment and cornea transplantation has poor prognosis for recurrence of the conjunctivalization. Current treatments of LSCD are aimed at replacing the damaged corneal stem cells in order to restore a healthy corneal epithelium. The autotransplantation of limbal tissue from the healthy, fellow eye is effective in unilateral LSCD but leads to depauperation of the stem cell reservoir. In the last decades, novel techniques such as cultivated limbal epithelial transplantation (CLET) have been proposed in order to reduce the damage of the healthy fellow eye. Clinical and experimental evidence showed that CLET is effective in inducing long-term regeneration of a healthy corneal epithelium in patients with LSCD with a success rate of 70%–80%. Current limitations for the treatment of LSCD are represented by the lack of a marker able to unequivocally identify limbal stem cells and the treatment of total, bilateral LSCD which requires other sources of stem cells for ocular surface reconstruction.

## 1. Introduction

The human cornea, which covers the anterior part of the ocular globe as a transparent dome, has an avascular, stratified, nonkeratinized squamous epithelium. It protects the eye from the outside environment, allowing vision at the same time. Total absence of blood vessels is essential for its transparency. Contiguous to the cornea and covering the sclera is the conjunctiva, which is rich in vessels and has a stratified columnar epithelium containing goblet cells.

**Figure 1:**
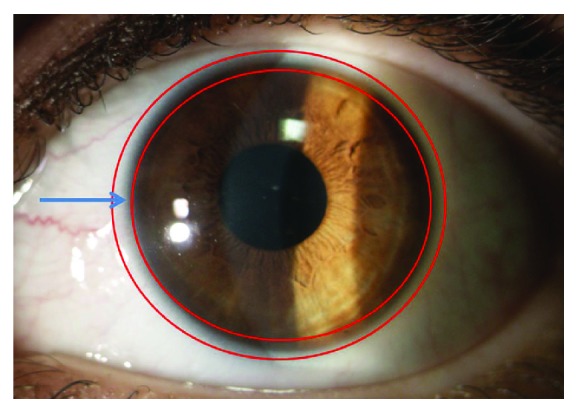
Limbal stem cells are located on the basal epithelium in the limbal crypts of the Vogt's palisades located in the narrow zone between the cornea and the bulbar conjunctiva (arrow).

The cornea maintains its transparency and continuously renews its epithelial surface by replacing, through a rapid turnover process, aged or injured epithelial cells. The presence of limbal stem cells guarantees epithelial cornea renewal. They reside on the basal epithelium in the limbal crypts of the Vogt's palisades located in the narrow zone between the cornea and the bulbar conjunctiva [1–4] (Figure 1). Limbal stem cells maintain a constant corneal cell number by retaining their capacity for self-renewal and, at the same time, by giving rise to transient amplifying cells (TAC). TAC are fast-dividing progenitor cells that provide the proliferative compartment of the limbal and corneal epithelia [5–10].

**Figure 2:**
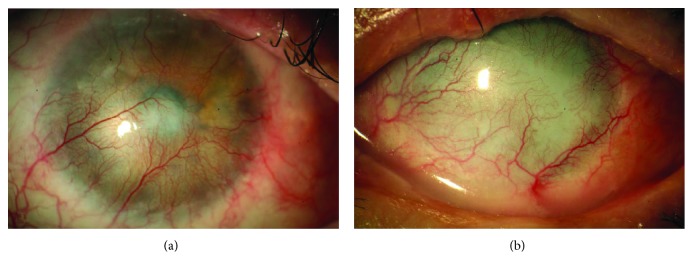
The loss of limbal stem cells results in cornea conjunctivalization (a) and pannus (b) with impairment of visual function.

All functional or anatomical conditions that damage limbal stem cells result in an impairment of corneal epithelial integrity and wound healing and cause a clinical entity named “limbal stem cell deficiency” (LSCD) [11, 12]. Several conditions have been reported to affect limbal stem cells leading to LSCD, including inherited (i.e., aniridia) corneal injuries (such as chemical burns, thermal injuries, multiple ocular surgeries, or cryotherapies) and chronic immune inflammatory diseases (i.e., Stevens-Johnson syndrome and ocular cicatricial pemphigoid) [13]. The partial or total loss of limbal stem cells leads to impairment of corneal epithelium turnover and healing resulting in a resurfacing of the cornea by invasion of the bulbar conjunctiva, known as “conjunctivalization” of the cornea. This process is an effective reparative mechanism to prevent secondary infections, deep ulceration, and perforation but leads to corneal vascularization and opacification, with consequent loss of vision [14, 15] (Figure 2). Currently, the diagnosis of LSCD is based on clinical history, recurrent or persistent epithelial defects, and on the presence of corneal conjunctivalization which can be confirmed using corneal cytological analysis by corneal impression cytology [16, 17]. The in vivo visualization of the limbal structures by in vivo confocal microscopy has also been proposed for the diagnosis of LSCD [18–20].

Corneal transplantation (keratoplasty) is a routine, effective, and safe surgical procedure to restore the corneal transparency in the presence of functional limbal stem cells. In fact, the clinical success of cornea transplantation relies on patients' own limbal stem cells, which generate the host-derived corneal epithelium. When the limbus is affected, a functional corneal epithelium can no longer be formed and the conjunctiva will invade the corneal surface with failure of the graft [10, 12]. To prevent corneal “conjunctivalization,” it is mandatory to replace a well-functioning limbus by means of transplantation of limbal stem cells.

Limbal stem cell transplantation (LSCT) has been developed for the treatment of corneal conditions associated with functional and/or anatomical loss of corneal epithelial stem cells to restore the damaged corneal surface allowing subsequent visual recovery. The first attempts to supply limbal stem cells consisted in autotransplantation of limbal/conjunctival tissue from the fellow eye [21, 22]. In the last decades, several novel techniques have been proposed including ex vivo expansion of human epithelial cells, mainly aiming at reducing the damage of the healthy fellow eye and at allowing the identification of stem cells in the tissue to be transplanted.

However, some important issues still represent a challenge, including the identification of positive stem cell markers to better isolate and characterize stem cells and the treatment of severe, total, bilateral LSCD.

This review reports the current state of the art on (i) the clinical application of human stem cells to treat corneal epithelial stem cell failure, (ii) the limitations of the existing procedures, and (iii) the future perspectives.

### 1.1. Location of the Epithelial Stem Cell Population in the Cornea and Candidate Markers

Although stem cell transplantations are currently introduced into clinical practice, a major challenge for stem cell biologists and clinicians is the identification of stem cells both in vitro and in vivo. No positive markers have been demonstrated to unequivocally identify limbal stem cells, and, currently, limbal stem cells can only be identified by indirect methods [23, 24]. From a translational point of view, it is crucial to know where the stem cells are located, which would allow defining precisely how and where to perform the biopsy for ex vivo stem cell expansion and finding markers for corneal epithelial stem cells that can validate the culture procedure.

It is widely accepted that in humans, the stem cells of the corneal epithelium are located only in the limbus region, segregated in specific structures known as limbal crypts in the palisades of Vogt [1, 25–28]. Several evidences support this location: (i) the identification of a population of label-retaining, slow-cycling cells located in clusters in the basal layer [1], (ii) lack of expression of corneal differentiation markers [29], (iii) ability of the limbal cells to generate holoclones (colonies of cells with high proliferative potential considered to be stem cells) [8, 30], (iv) impaired corneal epithelial regeneration when the limbus is damaged [12], and (v) restoration of the corneal surface in LSCD after limbal grafts or ex vivo limbal stem cell transplantation [21, 31, 32]. Candidate markers for limbal stem cells may be divided in (i) negative markers, including molecules that are expressed in the differentiated epithelium of the suprabasal layers of the limbus and in the central cornea, and (ii) positive markers, including molecules that are expressed by epithelial cells with high proliferative potential and specific clonogenic characteristics, located in clusters of cells in the basal layer of the limbus (Table 1). Negative markers include cytokeratins (CKs), such as CK3 and CK12 that are specifically expressed in the differentiated epithelium of the cornea and CK19 that is specifically expressed by the differentiated conjunctival epithelium [28, 29]. Evaluation of CK3/CK12 and CK19 expression is currently used to confirm the diagnosis of LSCD by corneal impression cytology. In fact, the presence of CK19 in the corneal cytology is an indirect demonstration of the presence of conjunctivalization [17]. Other negative markers include Desmoglein-3 and Connexin 43 (Cx43) and Connexin 50 (Cx50), both expressed in the corneal epithelium and absent in the basal limbal epithelium [23, 33–35].

Several other molecules have been proposed as putative positive markers, but this is still an area of controversy and conflicting results have been reported. Immunohistochemical studies showed that integrin *β*1 is expressed by both basal cells of the limbus and cornea and that integrin *α*9 is expressed at limbus basal epithelium by TAC [23, 28, 32]. Positive staining for high-affinity nerve growth factor receptor tyrosine kinase A (TrkA) was also demonstrated at the basal cell layer of ocular surface epithelia, with the highest intensity noted at the limbus [36]. Similarly, *α*-enolase and vimentin have been proposed as markers for limbal stem cells but they are expressed by the majority of basal limbal cells and lack of specificity for stem cell identification [28, 37].

The ATP-binding cassette transporter ABCG2, a member of the ABC transporters, has been proposed as a potential marker of a wide variety of stem cells including limbal stem cells [23, 38], since it is expressed in certain basal cells of human corneal limbus and absent in the suprabasal layer as well as in the central corneal epithelium. However further studies showed that; ABCG2+ cells are not associated with high colony forming efficiency [39]. More recently, the ATP-binding cassette transporter ABCB5, first demonstrated as a marker for skin progenitor cells and human malignant melanoma stem cells [40], has been proposed as a functional marker for stem cells of the corneal epithelium. ABCB5+ cells have been shown in the majority of label-retaining cells of the basal layer of the mouse limbus but not in the central cornea. Similarly, in the human eyes, ABCB5+ cells were located in the basal layer of the limbal epithelium and coexpressed ΔNp63*α*—a known regulator of regenerative potential in epithelial stem cells, including human limbal stem cells [41]—which was not expressed in ABCB5 cells. Moreover, grafts containing ABCB5+ cells were able to restore the corneal transparency and provided a stable, well-differentiated CK12+ epithelium [42]. The observation that ABCB5 knockout littermates develop a corneal epithelium that retains the capability of repairing central corneal injuries, however, suggests that corneal abnormalities arising from ABCB5 deficiency could be ascribed to the antiapoptotic role of the protein and that ABCB5 may play a role in regulating homeostasis of the limbal/corneal epithelium. The compensatory proliferation of nonapoptotic, basal keratinocytes could explain the observations regarding proliferation and cellularity in ABCB5 knockout mice, as well as the decreased number of label-retaining cells. The corneal opacification observed in extreme experimental conditions, such as the transplantation of cultured grafts made from ABCB5 cells, might reflect the loss of such a compensatory mechanism due to the transplantation of a pure population of ABCB5 cells [42].

To date, the most promising limbal stem cell marker candidates are C/EBP*δ*, Bmi1, ΔNp63*α*, and Notch-1 (Table 1). Immunohistochemical studies showed that transmembrane receptor Notch-1 is expressed by limbal basal epithelium and is colocalized with some ABCG2-positive cells suggesting that it may represent a potential marker for limbal stem cells [43–45]. The p63 nuclear transcription factor, a p53 homologue, was proposed as a marker for limbal stem cells; in fact, it represents an essential determinant of the proliferative potential of stem cells in stratified epithelia [28, 41, 46–49]. Experimental studies demonstrated that ablation of the p63 gene results in the absence of stratified epithelia [41, 50, 51] and mutations of p63 gene cause disorders of the epithelia development [51, 52]. In 2001, Pellegrini et al. demonstrated that p63 was expressed by basal limbal epithelial cells but not by human corneal epithelium [23, 41]. Ex vivo studies also showed that p63 is more expressed by smaller clonogenic cells than larger cells [53]. In vitro studies by Western blot demonstrated that holoclones contained high levels of p63 but not paraclones (colonies of cells with poor proliferative capacities) and that limbal basal cells expressing p63 also expressed the cell proliferation marker nuclear antigen (PCNA) [41, 46]. However, other studies detected positive staining for p63 in the limbal region and also among basal cells of the corneal epithelium, suggesting that basal p63-positive cells may include not only corneal epithelial stem cells but also cells in a proliferative state, such as TAC [28, 54]. The discrepancies in p63 expression detection may be due to differences in technical procedures, cross-species p63 expression pattern variations, and the use of a pan-p63 antiserum. In fact, limbal and corneal keratinocytes may contain different ΔN isoforms of p63 in different conditions. Specifically, the tumor protein p63 gene may generate two different premessenger RNAs, TAp63 and ΔNp63, and alternative splicing of each transcript produces *α*, *β*, and *γ* isoforms [32, 46]. It has been showed that ocular keratinocytes may contain all the ΔN isoforms and that ΔNp63*α* is detected at the limbus but not on the corneal epithelium in a healthy ocular surface while all isoforms, ΔNp63*α,* ΔNp63*β*, and ΔNp63*γ*, are expressed during activation of cornea wound healing and correlate with limbal cell migration and corneal regeneration and differentiation [46, 55]. The truncated dominant-negative ΔNp63 isoform of p63 is highly expressed in basal cells of many human stratified epithelia [51, 56]. Barbaro et al. demonstrated that cells coexpressing C/EBP*δ*, Bmi1, and ΔNp63*α* in vivo identified mitotically quiescent limbal stem cells and that, in vitro, these markers identified holoclone-forming cells, but not those forming meroclones and paraclones [39, 55, 57]. Barbaro et al. also suggested that in human limbal stem cells, proliferation potential relies on the expression of ΔNp63*α*, whereas self-renewal also requires C/EBP*δ*. In fact, C/EBP*δ* is able to induce mitotic quiescence and self-renewal of limbal stem cells and, at the same time, to positively regulate the expression of ΔNp63*α*, which sustains the proliferative potential of stem cells. After corneal injury, inactivation of C/EBP*δ* releases ΔNp63*α* + limbal stem cells and induces ΔNp63*α*-dependent limbal stem cell proliferation, with consequent migration and differentiation (associated with expression of ΔNp63*β* and ΔNp63*γ*) to regenerate the cornea [55]. Moreover, long-term stability of cultivated limbal stem cell transplantation was statistically associated with the percentage of p63-bright holoclone-forming stem cells in culture. Cultures in which p63 cells accounted for more than 3% were associated with a successful transplantation rate close to 80%. In contrast, cultures with less than 3% were associated with poor results, with successful transplantation in only 10% of patients [32].

### 1.2. Limbal Stem Cell Transplantation

The loss of limbal epithelial stem cells allows the conjunctival epithelium to invade the cornea. As a consequence, patients experience visual impairment and recurrent and/or persistent epithelial defects associated with chronic inflammation, discomfort, and pain [13]. Corneal impression cytology is currently used to identify goblet cells and/or cytokeratin 19 expression to confirm the presence of conjunctival epithelium and aid the diagnosis of corneal conjunctivalization [16–18].

Surgical approaches such as amniotic membrane transplantation may be useful in patients with partial and mild LSCD [13, 58–62]. However, they are deemed to fail in more severe cases of LSCD, which require the restoration of limbal stem cells before any other surgical procedure, to obtain long-term results [62]. In fact, limbal stem cell transplantation has shown to improve the prognosis of a subsequent keratoplasty [2, 63].

**Figure 3:**
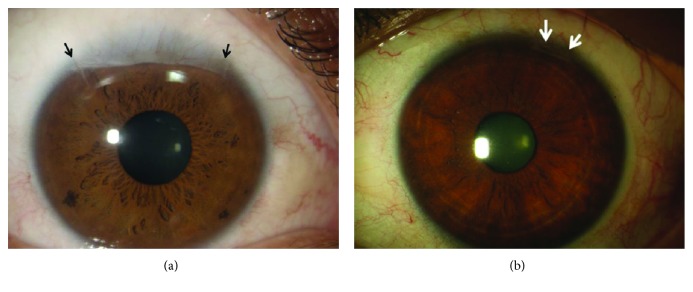
The limbal scar of the healthy donor eye after limbal biopsy to perform limbal autotransplantation (a) is larger than that after limbal biopsy to perform cultivated limbal epithelial transplantation (b).

Limbal stem cell deficiency has been successfully treated for years by directly grafting a portion of the healthy limbal tissue taken from the contralateral eye in unilateral cases [14, 21]. However, some concerns exist regarding potential donor eye risks [63]: although few reports have shown consequences related to harvesting [64], patients are often unenthusiastic about having the “good” eye touched, together with the great responsibility felt by surgeons. Moreover, further harvesting of the limbus following possible failure is not advisable (Figure 3(a)).

Recently, Sangwan et al. proposed the “simple limbal epithelial transplantation” (SLET) for unilateral LSCD, in which a small limbal biopsy from the contralateral healthy eye is extracted and then divided into 8–10 pieces and then placed on top of fresh human amniotic membrane already transplanted in the diseased cornea with fibrin glue [65]. This technique has been recently modified by using a cryopreserved amniotic membrane in a double layer that sandwiches the limbal cells, and it was approved by the FDA for clinical use [66]. Some concerns still exist regarding (i) the mechanism by which the biopsies work, (ii) reproducibility, and (iii) the long-term efficacy of the procedure. First of all, it is not clear if the small biopsies must be integrated and remain on the corneal surface forever, thus providing a stable source of corneal epithelium, a sort of “ectopic limbus,” or whether they should provide stem cells that will migrate onto the recipient cornea and repopulate the limbus. We should bear in mind that the migration process promotes stem cell differentiation and it has not yet been proven whether TA cells can redifferentiate into a stem cell state ([67]; Di [68]). Moreover, the amniotic membrane (AM), on which biopsies are glued, can both prevent and promote the correct engraftment and survival of the stem cells. The AM can integrate or be digested, thus affecting the fate of the biopsies. It has been shown that AM promotes the differentiation of limbal stem cells and AM itself has healing and regenerative properties [69]. It has also been shown that partial limbal stem cell deficiency can be treated with corneal pannus removal and AM grafting through in vivo recovery rather than stem cell engraftment [58]. However, SLET is a rapid and easy surgical procedure with the advantage of being low cost which made this technique of particular relevance in developing countries.

### 1.3. Development of Ex Vivo Limbal Stem Cell Transplantation

**Figure 4:**
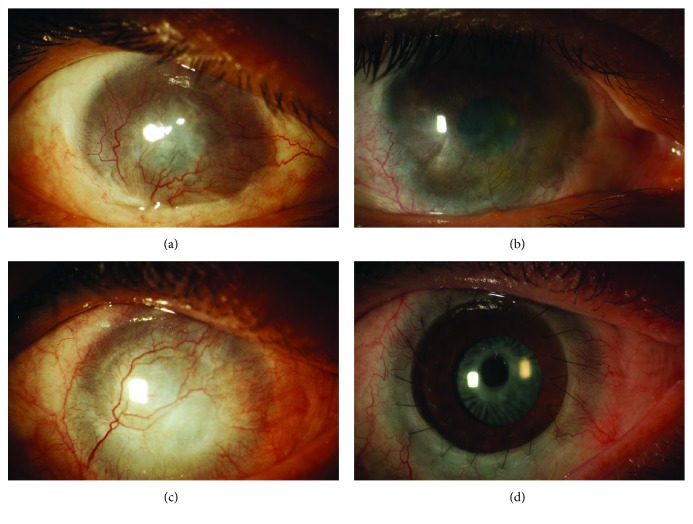
LSCD caused by chemical burn (a). Twelve months after cultivated limbal epithelial transplantation (b), the cornea showed cornea epithelial integrity, decrease of superficial neovascularization, and improvement of cornea transparency. Patients with LSCD after chemical burn with involvement of corneal stroma (c). The presence of corneal scarring required a keratoplasty after CLET to restore visual acuity (d).

To overcome risks for the donor eye, much effort has been made to develop a technique to reduce biopsy dimensions using cell expansion in culture. In 1997, Pellegrini et al. showed that autologous cultivated limbal epithelial transplantation (CLET) obtained from a 1 mm^2^ limbal biopsy included stem cells and restored the corneal surface in two patients with complete loss of the corneal-limbus epithelium [31] (Figure 3(b)). Subsequent studies showed that CLET was effective in inducing long-term regeneration of a healthy corneal epithelium in patients with LSCD due to chemical or thermal burns [70]. When the injury damaged only the ocular surface epithelium, including the limbus but sparing the corneal stroma, CLET was sufficient to restore corneal integrity and improve visual acuity; when the corneal stroma was involved by the injury, the corneal scarring required a subsequent keratoplasty for visual recovery [70] (Figure 4). The culture procedure was then standardized [8, 71], and to date, more than 270 grafts have been transplanted in different ophthalmological centres throughout Italy, with long-term stability reported in more than 150 patients and with a success rate in 70%–80% of cases [32, 72]. More than 10 years of follow-up confirmed the long-term integrity of the engrafted epithelium. The use of ex vivo-expanded autologous human corneal epithelial cells containing stem cell transplantation (Holoclar®) has been approved in 2015 by EMA for the treatment of patients with moderate or severe LSCD caused by burns.

Ex vivo limbal grafting represents an exciting innovation because it might have several advantages compared with the previously used technique of directly grafting limbal tissue: (i) fewer risks for the donor eye, (ii) possibility to treat bilateral LSCD when a spared part of the limbus, albeit small, is present, (iii) possibility of regrafting after failure, (iv) cells can be frozen and stored, allowing additional transplantation or banking if required, (v) association with gene therapy, and (vi) proof of concepts to use another cell source to treat total bilateral disease.

A successful LSCT mainly depends on a correct diagnosis and management of the patients. It is worth to note that LSCT is currently contraindicated in severe dry eye conditions, in which transplanted living tissue does not survive due to the alteration of the ocular surface microenvironment. In addition, LSCT is also contraindicated in the presence of active inflammation in bilateral diseases such as Stevens-Johnson syndrome, ocular cicatricial pemphigoid, EEC syndrome, and graft versus host disease [73]. Currently, it has been clearly demonstrated that autologous cultured limbal stem cells transplantation is effective in corneal epithelium restoration in patients with LSCD after chemical/thermal burns [32]. In these patients, the cytological diagnosis should be confirmed one year after injury, which is the time for a complete renewal of the corneal epithelium. Evidence showed that after cultured LSCD the transplanted stem cells multiply, migrate, and differentiate to regenerate the corneal epithelium and to replace lost limbal stem cells [32, 74]. The engrafted stem cells showed to maintain their self-renewal capacity, as demonstrated by their ability to regenerate a normal corneal epithelium after corneal transplantation performed 12–24 months after LSCT [32]. To improve the clinical outcome of LSCT, the proper selection and preparation of the recipient eye are of primary importance. In fact, failures of LSCT have been associated not only with the severity of the ocular damage but also with the severity of tear film impairment and presence of inflammation which cause changes of the ocular microenvironment [75]. As consequence, in patients with chemical burns, it is fundamental to restore eyelid morphology and function and to perform ocular surface reconstruction and improve tear film distribution before stem cell transplantation [32, 63, 76].

It is worthy of note that autologous limbal grafts may be performed in unilateral LSCD or in bilateral LSCD with spared portions of the healthy limbus that can be used as donor tissue for ex vivo expansion; however, in bilateral, total LSCD (when the limbus is completely destroyed in both eyes), limbal tissue from a deceased donor or from a living relative can be used or an alternative source of human stem cells, such as bone marrow and embryonic, oral, or skin epithelium, has been proposed [63, 77].

### 1.4. Ex Vivo Expansion and Carriers for the Cultivated Stem Cells

Various protocols for the cultivation of limbal stem cells for transplantation have been proposed, including methods to extract cells from biopsy (mechanical disruption or enzymatic dissociation), different substrates and carriers (fibrin sheet, amniotic membrane, contact lenses, and collagen), and the presence of animal-derived or xeno-free components in the system (medium and feeder layer) [78, 79]. Although good clinical outcomes have been reported with several different culture procedures, few studies have evaluated the clonal characteristics and proliferative potential of the cultivated cells. When dealing with stem cell-based therapies, it should be mandatory to demonstrate the presence, survival, and concentration of stem cells in culture and in the graft and validate the procedure under GMP conditions [8, 70].

We previously showed, using proliferative potential and clonal analysis, that from a 1-2 mm^2^ limbal biopsy, it was possible to isolate cells with stem cell characteristics that maintain their stemness once transferred onto a fibrin layer used as a carrier [8]. We also showed that autologous limbal stem cells, cultivated on fibrin and 3T3 feeder layers, maintain their properties and are able to restore corneal integrity in severe limbal stem cell deficiency [70]. Keratinocytes' culture was originally developed for epidermal keratinocytes and has been used in the last two decades for restoration of the corneal surface on hundreds of patients with LSCD with no adverse effects [80].

We later confirmed the long-term stability of the results, up to ten years, and validated the procedure, comparing clinical results with the expression of ΔNp63*α* in culture [32, 46, 72]. Success was statistically associated with the percentage of p63-positive cells in culture. Cultures in which p63 cells accounted for more than 3% were associated with a successful transplantation rate close to 80%. In contrast, cultures with less than 3% were associated with poor results, with successful transplantation in only 10% of patients [32].

## 2. Clinical Results

Since the first report in 1997 by Pellegrini et al., the efficacy of CLET to regenerate the ocular surface has been demonstrated in many reports [31, 81–83]. Baylis and colleagues recently reviewed the outcomes of cultured epithelial cell therapy over the past 13 years [63]. They evaluated 28 case report studies. These studies were very heterogeneous in terms of inclusion criteria, protocols, and assessment, and only 5 out of the 28 included more than 20 patients. Regarding the cause, bilateral chronic inflammatory diseases (Stevens-Johnson syndrome, cicatricial pemphigoid) were mixed together with congenital bilateral aniridia and posttraumatic unilateral and bilateral ocular burns, making the final conclusion very difficult to be drawn. The overall success rate was 77% for autografts and 73% for allografts, where the result of allografts was unexpected and a little surprising. The authors highlighted that different studies used different outcome parameters to evaluate the success of the procedures. In fact, some studies considered success the clinical evidence of an improved corneal surface, whereas others considered success more objective parameters such as visual acuity [63]. Similarly, Zhao et al. recently published a systematic review on the results of CLET using amniotic membrane as substrate for LSCD. They evaluated 18 studies with a follow-up period ranging from 1 to 118 months and reported a success rate of 67% and vision improvement of 62% without a difference between autograft and allograft [84].

In the literature, contrasting results have been reported on the use of allogeneic keratolimbal grafts: both clinical successes and failures have been observed in the presence of systemic immunosuppressive therapy [85, 86], while positive clinical results have been reported in the absence of immunosuppression [87, 88] and/or in the absence of allogeneic cell survival. A recent systematic review on clinical outcomes of keratolimbal allograft in LSCD after severe corneal chemical injury evaluated six nonrandomized, controlled studies and reported a best corrected visual acuity ≥ 20/200 in 69% (20/29) of the eyes at the last follow-up examination (mean follow-up range 6.2–114 months). The authors concluded that the quality of the evidence to support the use of keratolimbal allograft in LSCD is low and further, standardized studies with long-term follow-up are needed [89–91]. In addition, different studies used different immunosuppression regimens after keratolimbal allograft; in fact, even if most studies used systemic cyclosporine, there is not a standardized immunosuppressive regimen for keratolimbal allograft [91]. In most cases, the interpretation of the results has been hampered either by the lack of a proper genetic evaluation of the presumptive long-term engraftment of allogeneic limbal grafts or by the inadequate length of the follow-up. In the absence of demonstrated surviving donor cells, a possible explanation for the clinical success is that the allogeneic limbal cells grafted might have induced modifications of the microenvironment and promoted proliferation of the patient's own dormant stem cells, which progeny gradually replaced donor cells. While they remained in situ in the injured eye, these limbal cells evidently could not generate corneal epithelium, either because of the lack of a microenvironment suitable for their multiplication or because of fibrotic obstruction to their migration over the cornea. This would explain the mixed population of donor and recipient corneal cells observed at a short-term follow-up. These findings are consistent with reports showing that the clinical improvement observed by means of allogeneic keratolimbal grafts does not necessarily correlate with long-term survival of donor cells [92]. Similarly, cultured allogeneic epidermal keratinocytes do not engraft permanently but strongly stimulate epidermal regeneration in partial-thickness skin burns, presumably by stimulating residual hair follicle stem cells [93].

Recently, the CLET procedure has been approved for clinical use in the European Union and it is subject to the stringent regulations of the European Tissues and Cell Directive regarding good manufacturing procedures (GMP). The first study published in Europe in compliance with European Union regulations and GMP rules was performed in 2008 by Shortt and colleagues [94], who used a limbal cell suspension culture system and amniotic membrane as the substrate for limbal stem cell transplantation in 10 cases (7 allogeneic and 3 autologous) with a 6-month follow-up. In this study, the success rate was 60%, evaluated by corneal impression cytology and by in vivo corneal confocal microscopy, and visual acuity improvement was observed in 70% of patients. Although no statistical analyses were done, these authors reported better results for allogenic than for autologous transplantation. Their results may be influenced by the possibility that allograft tissues derived from donor cadavers were larger than the autologous tissue. In a subsequent study, our group reported a success rate of 68% after one autograft, up to a final successful clinical outcome of 76% after regrafting in 11 eyes, using fibrin-cultured autologous CLET in 107 cases, with a mean follow-up of 3 years [32]. Recently, Zakaria et al. reported the results of 15 autologous and 3 allogeneic (2 from HLA-matched living related donors and 1 from a cadaveric donor) xeno-free CLET using a human amniotic membrane support with a mean follow-up of 24 months. The grafts were assessed for the presence of progenitor cells, and the predominant phenotype (>50%) consisted of small cells positive for ∆Np63, CK14, and ABCG2 and negative for CK3/12 and desmoglein 3. They reported a clinical success rate of 67% defined as persistent continuous epithelial surface and a significant decrease of corneal neovascularization [95].

A 10-year retrospective study on xeno-free autologous CLET on a denuded amniotic membrane by Sangwan et al. on 200 patients with unilateral total LSCD due to chemical burns reported a 71% success rate evaluated on clinical grounds and a visual gain of two lines in 60.5% of cases, with a mean follow-up of 3 years [74]. They used sutures or fibrin glue and performed concomitant symblepharon surgery in 45% of cases and keratoplasty in 5% of them. The same group later reported a worse prognosis when keratoplasty was performed at the same time as autologous CLET than when CLET was done first and keratoplasty at least 6 weeks later [96].

### 2.1. Transplantation from Other Sources of Mucosal Epithelial Cell Transplantation

As previously mentioned, total, bilateral LSCD still represents a challenge for limbal transplantation and other sources of stem cells have been considered, including cultured oral mucosal epithelium transplant (COMET) [97–99] Nakamura et al. first proposed in 2003 the transplantation of cultured oral epithelium to restore the ocular epithelial surface in rabbits [100]. Oral and corneal epithelia have similar phenotypes, and the autologous epithelium showed a lower risk of immunologic rejection as compared to allotransplants. Subsequent studies showed that oral epithelium cells have a low stage of differentiation combined with fast cell turnover, need less time to grow in culture, and do not undergo keratinization [101]. Transplantation of cultured oral mucosa cells for human injured ocular surface reconstruction was turned into practice by several groups [102–105]. Nakamura and Kinoshita reported the development of neovascularization in patients treated with COMET, probably due to the lack of antiangiogenic factors [106]. Satake et al. reported a success rate of 65% at 1 year, 59% at 2, and 53% at 3 years after COMET in 40 eyes with total limbal stem cell deficiency due to Stevens-Johnson syndrome, chemical or thermal burns, and ocular cicatricial pemphigoid, evaluated with a mean follow-up of 25 months [107]. Recently, Sotozono et al. reported an improvement of visual acuity in 48% of 46 eyes with total LSCD after COMET, with a median follow-up of 28 months [98].

Other cell sources such as human embryonic stem cells, skin epidermal stem cells, hair follicle stem cells, bone marrow-derived mesenchymal stem cells, and immature dental pulp stem cells are being tested in experimental studies for their potential use for ocular surface reconstruction [82]. However, further experimental evidence is needed to demonstrate their efficacy in LSCD [108].

### 2.2. Future Perspectives and Conclusions

Several surgical procedures have been proposed for the treatment of LSCD in order to restore the limbal stem cell reservoir. After more than 40 years of studies, no consensus on guidelines for the management of this challenging condition has been reached. This is mainly due to heterogeneity of patient population, different follow-up periods, and outcomes, used in the clinical studies. In fact, while improvement of quality of life, visual acuity, or ocular surface may be considered a clinical success, to define a success of LSCT is necessary to formally demonstrate the long-term presence of the transplanted stem cells which is currently made by identification of corneal epithelium using corneal impression cytology.

The introduction of CLET for the treatment of patients with LSCD has represented a breakthrough for the management and outcome of this condition. The improvement in the knowledge of stem cell and corneal epithelium physiology, the setting up of culture methods able to maintain stemness in culture, and the development of surgical procedures to transplant ex vivo corneal epithelium sheets have defeated a challenging disease such as LSCD [82]. Two decades of experimental and clinical studies resulted in 70–80% success rates, and an improvement can be expected by identification of specific stem cell markers allowing a better biopsy procedure, sheet selection, and potentially enrichment of stem cell concentration.

Moreover, cultured limbal stem cell transplantation has several potential future improvements, such as selection of alternative carriers, gene therapies, and use of an alternative source of stem cells. These improvements might expand the clinical use of stem cells over LSCD for the treatment of genetic diseases, such as corneal dystrophies, or for performing a simultaneous transplantation of stem cells and corneal graft.

The main problems are related to bilateral LSCD without any spared limbal area which does not allow obtaining autologous limbal stem cells to be transplanted. Use of alternative sources of stem cells, such as oral mucosa, bone marrow-derived mesenchymal stem cells, or iPS cells, is under evaluation as alternative to limbal allografts obtained from cadaver or living relatives [91, 109, 110]. As alternative, implantation of an artificial cornea (keratoprosthesis) has been proposed for treatment of bilateral LSCD [111]. Encouraging results have been described with the Boston keratoprosthesis type I [112]. Recently, a systematic review demonstrated that 64% of patients with LSCD after chemical burns implanted with Boston keratoprosthesis type I showed a visual acuity of more than 20/200 and a device retention in 89% of cases, after 2 years of follow-up [113].

Future improvement of the ophthalmic use of stem cells includes (i) preparation of an “ex vivo cornea” composite by stem cells seeded with other cells, such as fibroblasts and endothelium, on a 3D scaffold and (ii) treating severe dry eyes by tissue engineering of the lacrimal gland and/or conjunctival tissue enriched with goblet cells.

## Figures and Tables

**Table 1:** Current putative markers of stem cell based on histological evidence.

Markers	Cornea		Limbus	
	Basal cells	Suprabasal cells	Basal cells	Suprabasal cells
CK3/CK12	++	++	--	+
CK19	-	-	+	-
CK5/CK14	+/-	-	+	+/-
Vimentine	-	-	++	+
*α* –enolase	+	-	++	+/-
Metallothionein	-	+	+/-	+
Connexin 43	++	+	-	+
Connexin 50	++	++	-	-
Desmoglein-3	-	+	-	+
E-cadherin	++	++	+/-	++
P-cadherin	+	-	+/-	-
Integrin *α*9	-	-	++	+/-
Integrin *β*1	++	+	+	+
Integrin *α*6	++	+	+/-	+
ABCG2	-	-	++	-
ABCB5	-	-	+	-
E-cadherin	++	++	+/-	++
P-cadherin	+/-	-	+/-	-
ΔNp63*α*	-	-	++	+/-
C/EBP*δ*	-	-	+	-
Bmi1	-	-	+	-
Notch 1	-	-	+	-
NGF-R TrkA	+	+/-	+	+/-
